# Progressive Medicine, Vol. IV., 1901

**Published:** 1902-07

**Authors:** 


PROGRESSIVE MEDICINE, VOL. IV., 1901. A Quarterly Digest of Advances, Discoveries and Improvements in the Medical and Surgical Sciences. Edited by Hobart Amory Hare, M. D., Professor of Therapeutics and Materia Medica in the Jefferson Medical College of Philadelphia. Octavo, handsomely bound in cloth, 400 pages, 13 illustrations. Per annum, in four cloth-bound volumes, $10.00. Lea Brothers & Co., Philadelphia and New York.

The articles contained in this volume are more than mere compilations. They constitute critical reviews and original papers of a very high order by men of established reputation.

Dr. Max Einhorn covers thoroughly the advances made during 1901 in both the medical and surgical treatment of diseases of the digestive tract and allied organs. Dr. Wm. T. Belfield discusses genito-urinary diseases, giving special attention to the subject of general infection by the gonococcus. Dr. Brubaker, under "Physiology," considers at length the therapeutic application of the gland extracts, etc. On "Hygiene," Dr. H. R. Baker writes an interesting section. He considers fully Dr. Koch's position on the transmissibility of bovine tuberculosis to man. Other contributors are E. Q. Thornton, on Therapeutics; J. R. Bradford, on Diseases of the Kidneys; and J. C. Bloodgood, on Anesthetics, Fractures, Dislocations, Surgery of the Extremities and Orthopedics.

